# Assessment of Knowledge, Practice and Attitude about Biomedical Waste Management among Healthcare Professionals during COVID-19 Crises in Al-Ahsa

**DOI:** 10.3390/healthcare9060747

**Published:** 2021-06-18

**Authors:** Sahbanathul Missiriya Jalal, Fahima Akhter, Amal Ismael Abdelhafez, Ahmed Mansour Alrajeh

**Affiliations:** 1 Department of Nursing, College of Applied Medical Sciences, King Faisal University, Al-Ahsa 31982, Saudi Arabia; falamin@kfu.edu.sa (F.A.); aabdelhameed@kfu.edu.sa (A.I.A.); 2 Department of Respiratory Therapy, College of Applied Medical Sciences, King Faisal University, Al-Ahsa 31982, Saudi Arabia; amalrajeh@kfu.edu.sa

**Keywords:** biomedical waste, healthcare, COVID-19, KPA, healthcare professionals

## Abstract

Biomedical waste (BMW) management is an essential practice of healthcare professionals (HCPs) for preventing health and also environmental hazards. Coronavirus disease (COVID-19) has become a global pandemic, posing significant challenges for healthcare sectors. A cross-sectional study was performed to assess the knowledge, practice, and attitude on BMW management among HCPs when taking care of patients with COVID-19 and associated with demographic variables. From Al-Ahsa healthcare sectors, 256 HCPs were selected randomly, of which 105 (41%) had excellent knowledge, 87 (34%) had good knowledge, and 64 (25%) had poor knowledge with a mean score of 13.1 ± 3.6. A higher mean score was (14.4 ± 3.2) obtained by physicians, and (13.6 ± 3.8) nurses than the other HCPs. Regarding practice, 72 (28.1%) HCPs used and discarded PPE while handling biomedical wastes. Additionally, 88 (34.4%) followed proper hand hygiene before and after each procedure and whenever needed. Physicians, nurses, and respiratory therapists had a more favorable attitude than other HCPs. There was a statistically significant association found among knowledge level and educational qualification (*p* < 0.0001), gender (*p* < 0.001), and work experience (*p* < 0.05). Emphasis is needed to train all HCPs regarding proper BMW management during this pandemic to prevent infection transmission.

## 1. Introduction

Biomedical waste (BMW) is healthcare wastes or hospital wastes generated from biological and medical activities, such as from the diagnosis, prevention, or treatment of diseases [1]. BMW must be managed by a safe and proper method, which should be known and practiced by every healthcare professional (HCP) to reduce the transmission of infection and to prevent various health and environmental hazards [2]. The international committee on the taxonomy of viruses termed the serious intense respiratory disease among people caused by coronavirus as coronavirus disease (COVID-19) on 11 February 2020. The World Health Organization (WHO) declared COVID-19 as the sixth public health emergency of international concern [3]. This severe pandemic crisis has been engendered a global health crisis in addition to its diverse impacts on the economy, society, and environment. Efforts taken to combat this pandemic have significantly increased the quantity of BMW generation. Moreover, the safe disposal of an increased quantity of BMW has been gradually posing a major challenge [4].

The possibility of confirmed COVID-19 cases and deaths has been increasing due to the continuous changing of the genome sequence of the coronavirus and its community transmission. Due to this pandemic, biomedical wastes (BMW) concerns not only to physicians or nurses, but to other HCPs such as pharmacists, technicians, interns, and therapists in hospitals and all healthcare sectors [5,6] as there was a paradigm shift in the form of the huge amount of wastes generated. There was an unexpected increase in the amount of disposable personal protective equipment (PPE), such as gloves, surgical mask, N-95 masks, air-purifying respirators, goggles, face shield, safety gowns or suits and shoe covers, as well as the use of plastic syringes and needles, high-flow nasal cannulas, and breathing circuits [7]. These have added a massive load to the waste management system.

In the eastern province of the Kingdom of Saudi Arabia, the generation rate of BMW is approximately 15 million tons per year with an average rate of 1.4 kg/capita/day and due to pandemics, this number is increasing dramatically [8]. BMW might act as a potential transmission source of infection and could likely be a hotspot to spread the infection, if waste is stored for a more extended period, especially in hospitals treating COVID-19 patients [9]. Again, as COVID-19 contaminated BMW is highly infective, it should not be disposed of in regular bins. Moreover, HCPs who handle such BMW should follow an adequate dress code, including wearing PPE, masks, splash-proof apron, gloves, gumboots, and safety goggles. As the virus has the potential ability to survive on face masks and gloves [10], adequate knowledge and proper techniques of handling these contaminated wastes and practice of safe disposal can protect the community from infection during this pandemic.

The World-Wide Fund for Nature (WWF), Italy, has stated that 10 million masks will be dispersed in the environment within a month, and if only one percent of the total masks are not disposed of properly, each mask weighing 4 g will add up to 40,000 kg of plastic [11]. In Indonesia, the scale of medical wastes reached 12,740 tons approximately 60 days after people were first infected by coronavirus [12,13]. Furthermore, India has been producing approximately six hundred metric tons of BMW daily, approximately 10% more than before [14]. Additionally, due to the lack of knowledge and poor practice on the proper disposal of medical waste at all levels of the healthcare sector, many countries dispose of their BMW in dustbins along with general household waste; some even reuse sharps items and syringes that eventually increase the risk of infection transmission, particularly in this current situation [15].

The improper practice of segregation at the site of origin has also been observed, which causes the mixing of infectious and non-infectious waste [16]. Color-coding schemes to segregate BMW should be strictly followed. Furthermore, wastes originating from COVID-19 wards should be collected, stored in separate records; and transported directly to treatment plants to avoid any cross-contamination [17,18]. In Europe, there is a trend among waste management sectors to provide separate collection services from households infected with COVID-19 and quarantine facilities [19].

Considering all these statistics, it is evident that a strategic guideline should be produced in Eastern province; Al-Ahsa, focusing on the current waste management knowledge, practice, and attitude (KPA) whilst handling, treating; and removing BMW produced during the identification, isolation; and management of COVID-19 patients. Additionally, the KPA should be updated from the regular processes of waste management by standardizing policy and organize awareness tracing programs on the BMW management system. The Al-Ahsa waste management authority should work together by formulating an extensive guideline involving the concerned ministry and other stockholders to fill and update the gaps in the knowledge, practice; and attitude among HCPs during this pandemic crisis. Therefore, the objectives of this study were to assess the knowledge, practice; and attitude regarding BMW management among HCPs during COVID-19 crises in Al-Ahsa healthcare sectors and to associate the knowledge level with the selected demographic variables.

## 2. Materials and Methods

### 2.1. Study Design

A descriptive design-based cross-sectional study was conducted among HCPs from the period of December 2020 to April 2021 in the governmental and non-governmental healthcare sectors caring for patients with COVID-19 in Al-Ahsa, Saudi Arabia.

### 2.2. Study Area and Setting

The study was conducted to assess the knowledge, practice and attitude on BMW management among various healthcare employees, who are involved in caring for COVID-19 patients in healthcare sectors, such as government and private hospitals, health centers; and polyclinics in Al-Ahsa, which is in the eastern province region of the Kingdom of Saudi Arabia.

### 2.3. Sample Size and Sampling Method

Considering the variables and outcome of the study, assuming the expected 50% of the study population had precise knowledge, good practice and a favorable attitude on BMW management, with an allowable margin error of 5%, at a 95% confidence interval, and accounting for the finite population for 922 HCP, a minimum sample size of 272 was calculated. After the randomization sampling, a total of 256 professionals from various fields were included in the data collection.

### 2.4. Inclusion Criteria

All HCPs including physicians, nurses, pharmacists, laboratory technicians (LTs), medical interns, nurse interns, X-ray technicians, and respiratory therapists (RTs), who were aged 20 years and above, both males and females, worked at government or private healthcare sectors in Al-Ahsa with a minimum 6 months professional experience and cared for COVID -19 patients, were included as study participants. HCPs who provided informed consent and available during data collection were included in the study.

### 2.5. Data Collection Tool and Procedure

The data were collected using a structured self-administered questionnaire (through Google Forms) and an observational checklist reviewing the literature and international BMW management guidelines. The questionnaire consisted of four sections. The first section included the socio-demographic information of HCPs. The second section included knowledge related to BMW management. The third section was an observational questionnaire assessing the practice of HCPs on BMW management; and the fourth section included the rating scale related to attitudes towards BMW management. The tool used that was developed in English. The pilot study was performed among 10 HCPs to test the tool. The reliability of the questionnaire was tested (r = 0.923) using Cronbach’s alpha. The time used to fill in the questionnaire ranged from 20 to 25 min. The objectives of the study were explained clearly to the participants before data collection. The privacy of respondents was assured by not asking their identity information such as their name, employee identity numbers, in the questionnaire. We used all data for the purpose of the research, and they were encrypted and stored electronically in a secure location, with a password used by the principal investigator to ensure privacy and confidentiality. Written informed consent was obtained from each participant, and then a structured questionnaire was distributed to collect all the data, except the practice tool. After the completion of the tool, using an observational method, the practice questionnaire was filled by data collectors.

#### 2.5.1. Demographic Information

The parameters of demographic data of HCPs included age, gender, educational qualification, occupation, professional experience; and working area.

#### 2.5.2. Knowledge Questionnaire

This tool consisted of 20 multiple choice questions, each of which had four options in which there was three incorrect with one correct option. The structured knowledge questionnaire was scored as either one for a correct response or zero for an incorrect response. The total knowledge score was summed and computed for analysis. The score interpretations were counted from 75% to 100% (15 to 20) as excellent knowledge, from 50% to 74% (10 to 14) as good knowledge; and below 50% (less than 10) as poor knowledge.

#### 2.5.3. Practice Questionnaire

The observation questionnaire was used to assess the practice of HCPs in healthcare sectors. It contained ten questions with the options “always”, “sometimes “, and “never”. The investigators observed the practice of HCPs and filled in the questionnaire directly. The overall practice was calculated and interpreted using the frequency distribution table in the results section.

#### 2.5.4. Attitude Scale

The questionnaire on attitude towards BMW management comprised ten questions. A five-point Likert scale of measurement was used to represent the scores; “strongly agree;” “agree;” “neutral;” “disagree;” and “strongly disagree”, which was scored as 5, 4, 3, 2; and 1 respectively and for negatively phrased statements, scores were reversely coded during the data entry period as 1, 2, 3, 4; and 5, respectively. The overall score of attitude was calculated by adding all scores of HCPs and the mean was computed by dividing the overall attitude score by the number of study participants (256). Finally, attitude scores below the mean and above or equal to the mean score were assigned for unfavourable and favourable attitudes, respectively.

### 2.6. Ethical Considerations

Ethical approval was obtained from the Research Ethics Committee, Deanship of Scientific Research, King Faisal University, Al-Ahsa, Saudi Arabia (HAPO-05-HS-003). All HCPs gave consent before participation and were informed about confidentiality, the lack of risk, anonymity; and voluntary participation. The research protocol was also approved by the King Fahad Hospital, Hofuf, Institutional Review Board, Saudi Arabia (H-05-HS-055) with reference number 55-35-2020. During data collection, after assessing the inclusion criteria, the objectives of the study and the research purposes were explained to all study participants clearly, and written informed consent was obtained. They were permitted to withdraw from the study at any stage according to their interest. The participants were assured that their data would remain confidential. This study was conducted by the Declaration of Helsinki and followed ethical principles.

### 2.7. Statistical Analysis

Statistical Package for Social Sciences (SPSS), (IBM Corp. Released 2012. IBM SPSS Statistics for Windows, Version 21.0. Armonk, NY, USA: IBM Corp.) was used to analyze the study data. The numbers and percentages were tabulated in the form of the frequency distribution, mean; and standard deviation calculated using descriptive analysis. Chi-square analysis was used to test the association between the knowledge of HCPs on BMW management and their demographic characteristics, and the *p* value was equal to 0.05 or less.

## 3. Results

### 3.1. Demographic Characteristics of the HCP

Out of 256 HCPs included in the analysis (Table 1), 123 (48.1%) were in the age of 20–30 years, and the majority of the participants, 172 (67.2%), were females. Most of the participants, 152 (59.4%), had bachelor’s degrees as their highest educational qualification. Few, 15 (5.9%), were doctorates. Regarding the occupational status of the HCPs, 57 (22.3%) were physicians, 92 (35.9%) were nurses, and 20 (7.8%) were RTs. Furthermore, 85 (33.2%) HCPs had a minimum of six months to one year of experience, 59 (23.1%) had 4 to 6 years of experience and 38 (14.8%) had 6 to 9 years of experience. Concerning the working area, most of the participants, 132 (51.6%), worked in a government hospital.

### 3.2. Knowledge Level of the HCPs on BMW Management

**Figure 1:**
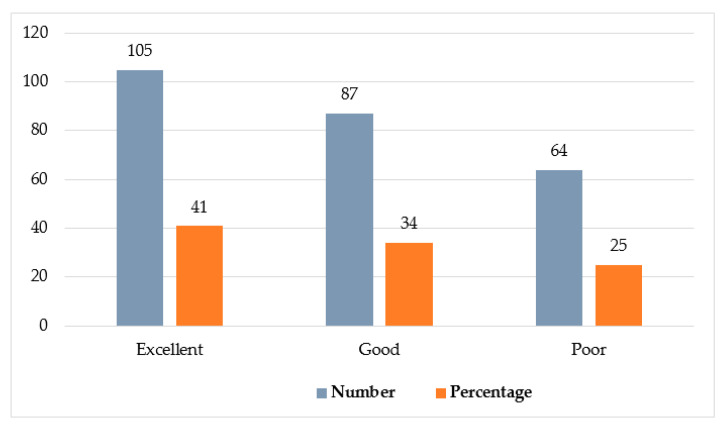
Knowledge level of HCP about BMW management.

The overall knowledge level of HCPs is shown in Figure 1, in which; 105 (41%) had excellent knowledge, 87 (34%) had good knowledge and 64 (25%) had poor knowledge. The descriptive statistical report of the knowledge level of HCPs is evidenced in Table 2. The overall mean score was 13.1 ± 3.6. A high mean score 14.4 ± 3.2 was obtained by physicians, then 13.6 ± 3.8 by nurses, 13 ± 3.8 by pharmacists, 13.1 ± 2.1 by LT, 12.8 ± 3.9 by interns (medical), 12.5 ± 3.4 by interns (nurse); and 13.1 ± 3.3 by RTs.

### 3.3. Practice of HCPs in BMW Management

The practice of HCPs in BMW management is reported in Table 3. Most of the HCPs 203 (79.3%), always followed the guidelines specified by the Ministry of Health (MOH) for BMW management. Approximately, 196 (76.6%) HCPs always adhered to the infection control policies while treating COVID-19 patients. Most of the HCPs, 163 (63.7%), sometimes used and discarded all PPE while handling BMW. Approximately, 177 (69.1%) HCPs followed the color coding of containers according to the type of waste during the disposal of BMW and 102 (39.8%) followed policies in separating the wastes into non-hazardous, hazardous; and sharp waste. Additionally, 181 (70.7%) maintained BMW records. Regarding preventing sharps related injury such as avoiding recapping used needles, 138 (53.9%) HCPs were cautious, and 192 (75%) HCPs prevented contamination while handling items of COVID-19 patients and other non-COVID-19 patients.

### 3.4. Attitude of the HCPs towards BMW Management

The results showed that 187 (73.1%) had a favorable attitude, and 69 (26.9%) had an unfavorable attitude towards BMW management. Among them, most of the physicians (89%) and nurses (78%) had a more favorable attitude than others. As shown in Table 4, 193 (75.4%) HCPs strongly agreed that the safe disposal of BMW was necessary for healthcare areas. Approximately 134 (52.3%) HCPs strongly agreed that BMW management required teamwork. However, only 63 (24.6%) strongly disagreed that BMW management created an extra burden on their work. Most of HCPs, 124 (48.3%), strongly disagreed that BMW management risked transmitting infectious diseases. However, 6 (2.3%) strongly disagreed that the segregation of hospital waste into different categories was time consuming. Approximately 112 (43.8%) HCPs strongly felt that PPE must be used while handling BMW, and 119 (46.5%) felt that decontamination and disinfection reduced infection. The majority, 141 (55.1%), strongly agreed that proper BMW management enhanced the quality assurance of healthcare sectors, and 128 (50%) felt strongly that upgraded knowledge on BMW management was mandatory.

### 3.5. Association of the Knowledge of HCPs on BMW Management with Demographic Variables

Table 5 shows that there was a significant association between the level of knowledge and three demographic parameters; namely gender (*p* < 0.001), educational qualification (*p* < 0.0001) and work experience (*p* < 0.05).

## 4. Discussion

Public health must be protected from environmental hazards by every healthcare sector through following proper BMW management. During this COVID-19 pandemic, many government agencies, including MOH, have published guidelines for the management of waste produced during the treatment, diagnosis; and isolation of COVID-19 patients. It must be managed properly to prevent the severe risk of contamination and disease transmission. Our study was conducted to assess the knowledge, practice; and attitude on BMW management among HCPs in the eastern region of Saudi Arabia. Our study reported that 41% had excellent knowledge, 34% had good knowledge and 25% had poor knowledge. This finding was supported by a study conducted in Saudi Arabia on the knowledge, attitude; and practices of healthcare workers regarding BMW of COVID-19 in the Aseer Region, where healthcare workers had sufficient knowledge on COVID-19 and infection control measures [20]. Another cross-sectional study was performed to analyze the knowledge, practices, and attitudes of healthcare workers regarding coronavirus disease 2019 (COVID-19) across 10 hospitals in Henan, China. In that report, 89% of HCPs had sufficient knowledge [21].

A survey study designed to investigate the knowledge, attitudes; and practices of doctors, nurses, laboratory technicians; and housekeeping staff, regarding medical waste management at a tertiary hospital in Gaborone, Botswana, proved that there was a significant agreement among the participants on the proper segregation of medical waste to be carried out at the point of generation, with a mean score 4.43 out of 5, and on the color-coding system, with a mean score of 4.59 out of 5 [22]. In the current study, the overall mean score was 13.1 ± 3.6 for the knowledge questionnaire regarding BMW management. An observational cross-sectional study was conducted on the awareness and practice of medical waste management among healthcare providers in National Referral Hospital, in which approximately 74.4% participants were aware of medical waste management, and 98.2% were aware of the importance of using proper PPE [23].

An observational study carried out to provide an overview of the management of BMW in a tertiary care teaching hospital showed that 30% to 35% of respondents did not practice this [24]. Another study evidenced that [25] regarding practice, 68% of HCPs knew that the most important step in waste management is waste segregation, and 82% of the participants working in this setup knew the different color-coded bins used for segregation [15]. In our study, most of the HCPs (79.3%) always followed the MOH guidelines for BMW management, and 69.1% of HCPs carried out the color coding of containers during the disposal of BMW according to the type of waste. Approximately 76.6% of HCPs always adhered to the infection control policies while treating COVID-19 patients. A study performed at the large hospitals in Bangalore; indicated that, although there was an absence of committees for infection control and hospital waste management, 20% of nursing homes had a policy for healthcare waste management [26]. 

Every HCP must be informed on the proper handling, disinfecting; and wearing of PPE. A study on the knowledge, attitude and practices of healthcare workers regarding BMW of COVID-19 in the Aseer Region showed a poor understanding of the protocols and policies of PPE disposal [20]. However, in this study, most of them, 163 (63.7%), sometimes used and discarded sometimes all personal protective equipment while handling biomedical waste, and 102 (39.8%) followed policies in separating BMW into non-hazardous, hazardous; and sharp waste.

A cross-sectional study conducted among healthcare personnel working at primary health centers; in Gujrat showed that the highest overall scores for attitudes to waste disposal were observed among housekeepers compared to physicians or LTs [27]. However, in our study, the results showed that 73.1% had a favorable attitude, and 26.9% had an unfavorable attitude towards BMW management. Among them, those with the highest number of favorable attitudes were physicians (89%) and nurses (78%). This was supported [22,28,29] by a study in India, at a tertiary level healthcare institution, where doctors (100%) were found to be more positive towards the need for actions for safe biomedical waste management than nurses (60%) and other healthcare workers [30].

A study performed in Alburaimi hospital, Oman, regarding the attitude of healthcare workers towards the safe management of BMW, proved that the majority of LTs (92.7%) considered BMW as an issue as compared to nurses (87.3%), doctors (80.5%); and housekeeping staff (80%), although it was statistically insignificant (*p* = 0.639). Moreover, a significantly higher percentage of nurses (92.7%) than doctors (83.2%); and LTs (64.3%), agreed that BMW management requires teamwork, and no single class of people was responsible this (*p* = 0.024) [31]. However, in this study, 75.4% of HCPs strongly agreed that the safe disposal of BMW was necessary for the healthcare areas. Approximately, 52.3% of HCPs strongly agreed that the BMW management required teamwork. However, only 24.6% strongly disagreed that BMW management created an extra burden on their work.

Research on attitude regarding BMW awareness proved that many of healthcare workers (93.3%–98.9%) were aware of improper waste management which was causing various health hazards; (79.8% to 97.9%), the importance of regular educational programs on BMW management; (75.7% to 82%), the amount of generated BMW in hospitals or clinics and (52.8% to 87.6%) that maintaining BMW records is mandatory in hospitals or clinics [32,33]. In this study, most HCP 48.3% disagreed strongly that BMW management was risks transmitting infectious diseases. However, the majority of HCPs (55.1%) agreed that proper BMW management enhanced the quality assurance of healthcare sectors and 50% strongly felt that upgraded knowledge on BMW management was essential.

Descriptive research was performed on the knowledge, attitude; and practices of healthcare staff regarding infectious waste handling at tertiary care health facilities in the metropolitan city of Pakistan, in which the sociodemographic information such as age, gender, level of education; and experience, when compared with the practices, was found to be statistically significant (*p* < 0.05) [34]. In our study, there was also a significant association between the level of knowledge and demographic characteristics, such as educational qualification (*p* < 0.0001), gender (*p* < 0.001); and work experience (*p* < 0.05). This impetuous COVID-19 situation changed healthcare systems, and the pandemic crisis forced many hospitals to reorganize their healthcare systems [35]. Hence, this study was performed to find the level of the knowledge, practice; and attitude of HCPs on BMW management during this pandemic.

This study also has some limitations. There was a chance for recall bias in this study due to memory recall for knowledge-related questions. However, randomization in the selection of samples was used to reduce the bias. The practice was observed directly, which could have been biased. The participating HCPs were mostly females which may have affected the association findings. We did not assess the culture and nationality of the participants, which we recommend in future studies. This study could be repeated as an interventional investigation with larger samples, including all kinds of healthcare workers.

## 5. Conclusions

HCPs are frontline workers in the COVID-19 crisis; they face a greater risk of contamination due to their direct contact with patients and specimens. In this situation, BMW must be considered a serious health concern. Accordingly, HCPs must have adequate knowledge regarding the proper handling of BMW, prevention of infection; and prevention of transmission of diseases. This study was intended to assess the KPA of HCPs on BMW management in this pandemic crisis. The present findings demonstrated the necessity to organize continuous training programs in the form of symposia, seminars; and workshops on BMW management to develop awareness among HCPs. A high level of practice regarding the proper handling of PPE is recommended in the present study. In the current scenario, training could be a key factor for HCPs for effective BMW management. Hence, the concerned authorities should assign significant importance to develop a nationally recognized standard guideline in all health sectors to manage BMW and reduce the risk of the pandemic spreading in the community.

## Figures and Tables

**Table 1:** Demographic characteristics of the HCP (n = 256).

Items		N	%
Age (years)	20–30 years	123	48.1
	31–40 years	83	32.4
	41–50 years	38	14.8
	More than 50 years	12	4.7
Gender	Male	84	32.8
	Female	172	67.2
Educational Qualification (Highest)	Diploma	28	10.9
	Bachelor	152	59.4
	Master	38	14.8
	Doctorate	15	5.9
	Others	23	9
Occupation	Physician	57	22.3
	Nurse	92	35.9
	Pharmacist	18	7
	Lab technician (LT)	22	8.6
	Intern (Medical)	21	8.2
	Intern (Nurse)	26	10.2
	Respiratory therapist (RT)	20	7.8
Professional Experience	6 months to 1 year	85	33.2
	1–3 years	53	20.7
	4–6 years	59	23.1
	6–9 years	38	14.8
	10 and above years	21	8.2
Working Area	Government hospital	132	51.6
	Private hospital	78	30.5
	Heath centre	24	9.3
	Polyclinic	22	8.6

N—number; %—percentage.

**Table 2:** Descriptive statistical report of knowledge of HCP about BMW management (n = 256).

HCPs	Physician	Nurse	Pharmacist	Lab Technician	Interns (Medical)	Interns (Nurse)	RT	Total
Count	57	92	18	22	21	26	20	256
Mean	14.4	13.6	13	13.1	12.8	12.5	12.3	13.1
Median	16	14	14	14	13	14	13	14
Largest	19	19	19	17	19	17	17	19
Smallest	8	7	7	9	7	8	8	7
SD	3.2	3.8	3.8	2.1	3.9	3.4	3.3	3.6
Variance	10.2	14.4	14.1	4.8	15.5	11.9	9.3	12.7

SD—standard deviation.

**Table 3:** Practice of HCP about BMW management *(n* = 256).

S. No.	Practice on BMW Management	Always	Sometimes	Never
		N (%)	N (%)	N (%)
1	Does she/he follow the guidelines laid down by Ministry of Heath for BMW management?	203 (79.3)	49 (19.1)	4 (1.6)
2	Does she/he adhere the infection control policy while handling COVID-19 patients?	196 (76.6)	52 (20.3)	8 (3.1)
3	Does she/he use all personal protective equipment while handling biomedical wastes?	72 (28.1)	163 (63.7)	21 (8.2)
4	Does she/he discard all personal protective equipment after handling biomedical wastes?	72 (28.1)	163 (63.7)	21 (8.2)
5	Does she/he follow proper hand hygiene before and after every procedure and frequently?	88 (34.4)	159 (62.1)	9 (3.5)
6	Does she/he follow colour coding of containers according to the type of wastes while for disposing BMW?	177 (69.1)	51 (19.9)	28 (10.9)
7	Does she/he follow policies separating BMW as non-hazardous, hazardous, and sharp waste in segregation?	102 (39.8)	82 (32.1)	31 (12.1)
8	Does she/he maintain BMW records?	181 (70.7)	42 (16.4)	33 (12.9)
9	Does she/he take care in preventing sharp related injury like avoid recapping used needle?	138 (53.9)	112 (43.8)	6 (2.3)
10	Does she/he prevent contamination while handling items of COVID-19 patients and other non-COVID-19 patients?	192 (75)	52 (20.3)	12 (4.7)

N—number; %—percentage.

**Table 4:** Attitude of the HCPs towards BMW management (n = 256).

S. No.	Attitude Questions	Strongly Agree	Agree	Neutral	Disagree	Strongly Disagree
		N (%)	N (%)	N (%)	N (%)	N (%)
1.	Safe disposal of BMW is necessary in health care areas.	193 (75.4)	34 (13.2)	25 (9.8)	3 (1.2)	1 (0.4)
2.	BMW management is a team work.	134 (52.3)	52 (20.3)	37 (14.5)	22 (8.6)	11 (4.3)
3.	BMW management creates extra burden on my work. *	31 (12.1)	42 (16.4)	78 (30.5)	42 (16.4)	63 (24.6)
4.	BMW management is risk to transmit any infectious diseases. *	15 (5.9)	27 (10.6)	44 (17.2)	46 (18)	124 (48.3)
5.	Segregate hospital waste into different categories is time consuming. *	126 (49.2)	31 (12.1)	68 (26.6)	25 (9.8)	6 (2.3)
6.	PPE is must while handling biomedical waste.	112 (43.8)	82 (32)	41 (16)	18 (7)	3 (1.2)
7.	Decontamination and disinfection reduces the infection.	119 (46.5)	68 (26.6)	51 (19.9)	13 (5)	5 (2)
8.	Use of colour code for segregation of wastes are must.	201 (78.5)	29 (11.4)	17 (6.6)	7 (2.7)	2 (0.8)
9.	Proper BMW management enhance the quality assurance of health care sectors.	141 (55.1)	76 (29.7)	21 (8.2)	11 (4.3)	7 (2.7)
10.	Upgrade knowledge on BMW management is mandatory.	128 (50)	53 (20.8)	31 (12)	29 (11.3)	15 (5.9)

* Negatively phrased statements and reversely scored.

**Table 5:** Association of the knowledge of HCPs with demographic variables (n = 256).

Demographic Variables		Excellent	Good	Poor	X^2^
Age (years)	20–30 years	45	40	38	X^2^ = 11.4833 *p* = 0.074539 NS
	31–40 years	30	34	19	
	41–50 years	22	11	5	
	More than 50 years	7	4	1	
Gender	Male	28	43	13	X^2^ = 14.0327 *p* = 0.000897 *
	Female	75	48	49	
Educational Qualification (Highest)	Diploma	1	10	17	X^2^ = 70.5972 *p*—0.00001 *
	Bachelor	50	66	36	
	Master	30	7	1	
	Doctorate	13	1	1	
	Others	9	11	3	
Occupation	Physician	31	18	8	X^2^ = 12.55807 *p* = 0.4019622 NS
	Nurse	31	38	23	
	Pharmacist	6	7	5	
	Lab Technician	12	6	4	
	Intern (Medical)	8	11	2	
	Intern (Nurse)	12	10	6	
	RT	6	9	5	
Professional Experience	I year	34	23	28	X^2^ = 19.6762 *p* = 0.011633 *
	1–3 years	16	22	15	
	4–6 years	20	26	13	
	6–9 years	23	10	5	
	10 and above years	10	10	1	
Working Area	Government hospital	46	59	27	X^2^ = 12.2509 *p* = 0.056599 NS
	Private hospital	34	23	21	
	Heath centre	12	6	6	
	Polyclinic	11	3	8	

X^2^—Chi-square test; * significant; NS—non-significant; *p* < 0.05.

## Data Availability

The data presented in this study are available within the article.

## Abbreviations

BMW	Biomedical waste
COVID-19	Corona virus disease-19
HCP	Health care professionals
IBM	International Business Machines Corporation
KPA	Knowledge, practice, and attitude
LT	Lab technicians
MOH	Ministry of Health
NS	Non-significant
PPE	Personal protective equipment
RT	Respiratory therapists
SD	Standard deviation
SPSS	Statistical Package for the Social Sciences
WHO	World Health Organization
WWF	World-wide Fund of Nature

## References

[1] Goswami M., Goswami P.J., Nautiyal S., Prakash S. (2021). Challenges and actions to the environmental management of Bio-Medical Waste during COVID-19 pandemic in India. Heliyon.

[2] Deress T., Hassen F., Adane K., Tsegaye A. (2018). Assessment of knowledge, attitude, and practice about biomedical waste management and associated factors among the healthcare professionals at Debre Markos town healthcare facilities, northwest Ethiopia. J. Environ. Public Health.

[3] Lai C.C., Shih T.P., Ko W.C., Tang H.J., Hsueh P.R. (2020). Severe acute respiratory syndrome coronavirus 2 (SARS-CoV-2) and coronavirus disease-2019 (COVID-19): The epidemic and the challenges. Int. J. Antimicrob. Agents.

[4] Rao D., Dhakshaini M.R., Kurthukoti A., Doddawad V.G. (2018). Biomedical Waste Management: A Study on assessment of knowledge, attitude and practices among health care professionals in a tertiary care teaching hospital. Biomed. Pharmacol. J..

[5] Musa F., Mohamed A., Selim N. (2020). Assessment of nurses’ practice and potential barriers regarding the medical waste management at Hamad medical corporation in Qatar: A cross-sectional Study. Cureus.

[6] Thind P.S., Sareen A., Singh D.D., Singh S., John S. (2021). Compromising situation of India’s bio-medical waste incineration units during pandemic outbreak of COVID-19: Associated environmental-health impacts and mitigation measures. Environ. Pollut..

[7] Shammi M., Behal A., Tareq S.M. (2021). The escalating biomedical waste management to control the environmental transmission of COVID-19 pandemic: A perspective from two south Asian countries. Environ. Sci. Technol..

[8] Yousefi M., Oskoei V., Jonidi Jafari A., Farzadkia M., Hasham Firooz M., Abdollahinejad B., Torkashvand J. (2021). Municipal solid waste management during COVID-19 pandemic: Effects and repercussions. Environ. Sci. Pollut. Res. Int..

[9] Ammendolia J., Saturno J., Brooks A.L., Jacobs S., Jambeck J.R. (2021). An emerging source of plastic pollution: Environmental presence of plastic personal protective equipment (PPE) debris related to COVID-19 in a metropolitan city. Environ. Pollut..

[10] Khoironi A., Hadiyanto H., Anggoro S., Sudarno S. (2020). Evaluation of polypropylene plastic degradation and microplastic identification in sediments at Tambak Lorok coastal area, Semarang, Indonesia. Mar. Pollut. Bull..

[11] Datta P., Mohi G.K., Chander J. (2018). Biomedical waste management in India: Critical appraisal. J. Lab. Physicians.

[12] Bhagawati G., Nandwani S., Singhal S. (2015). Awareness and practices regarding bio-medical waste management among health care workers in a tertiary care hospital in Delhi. Indian J. Med. Microbiol..

[13] Mihai F.C. (2020). Assessment of COVID-19 waste flows during the emergency state in Romania and related public health and environmental concerns. Int. J. Environ. Res. Public Health.

[14] Yadavannavar M., Berad A.S., Jagirdar P. (2010). Biomedical waste management: A study of knowledge, attitude, and practices in a tertiary health care institution in bijapur. Indian J. Community Med..

[15] Parida A., Capoor M.R., Bhowmik K.T. (2019). Knowledge, attitude, and practices of bio-medical waste management rules, 2016; bio-medical waste management (amendment) rules, 2018; and solid waste rules, 2016, among health-care workers in a tertiary care setup. J. Lab. Physicians.

[16] Shaheen T., Ghani M., Kausar S. (2020). Gauging the effectiveness of training sessions among nurses regarding biomedical waste management: A quasi-experimental study from a developing country. Cureus.

[17] Rashidian A., Alinia C., Majdzadeh R. (2015). Cost-effectiveness analysis of health care waste treatment facilities in Iran hospitals; a provider perspective. Iran. J. Public Health.

[18] Singh S., Dhillon B.S., Nityanand, Shrivastava A.K., Kumar B., Bhattacharya S. (2020). Effectiveness of a training program about Cat a tertiary care teaching institute of North India. J. Educ. Health Promot..

[19] Nghiem L.D., Morgan B., Donner E., Short M.D. (2020). The COVID-19 pandemic: Considerations for the waste and wastewater services sector. Case Stud. Chem. Environ. Eng..

[20] Alshahrani N.Z., Alshaiban H.M., Alarbash H.A., Mahmood S.E., Aljunaid M.A., Albeshry A.M., Sayyad Y. (2021). Knowledge, Attitude and Practices of Healthcare Workers regarding Bio-medical Waste of COVID-19 in Aseer Region, KSA. Int. J. Pharm. Res..

[21] Zhang M., Zhou M., Tang F., Wang Y., Nie H., Zhang L., You G. (2020). Knowledge, attitude, and practice regarding COVID-19 among healthcare workers in Henan, China. J. Hosp. Infect..

[22] Mugabi B., Hattingh S., Chima S.C. (2018). Assessing knowledge, attitudes, and practices of healthcare workers regarding medical waste management at a tertiary hospital in Botswana: A cross-sectional quantitative study. Niger J. Clin. Pract..

[23] Letho Z., Yangdon T., Lhamo C., Limbu C.B., Yoezer S., Jamtsho T., Chhetri P., Tshering D. (2021). Awareness and practice of medical waste management among healthcare providers in National Referral Hospital. PLoS ONE.

[24] Pandey A., Ahuja S., Madan M., Asthana A.K. (2016). Bio-medical waste management in a tertiary care hospital: An overview. J. Clin. Diagn. Res..

[25] Ilyas S., Srivastava R.R., Kim H. (2020). Disinfection technology and strategies for COVID-19 hospital and bio-medical waste management. Sci. Total Environ..

[26] Chethana T., Thapsey H., Gautham M.S., Sreekantaiah P., Suryanarayana S.P. (2014). Situation analysis and issues in management of biomedical waste in select small health care facilities in a ward under Bruhat Bengaluru Mahanagara Palike, Bangalore, India. J. Community Health.

[27] Sunmeet G.A., Gangawane A. (2017). Knowledge attitude and practices of healthcare personnel towards bio-medical waste disposal management at Arbor Biotech Limited Mumbai. Int. J. Innov. Res. Sci. Eng..

[28] Aanandaswamy T.C., Rajappa G.C., Venkatachala N., Kamath R. (2019). Assessment of knowledge, attitude, and practices regarding biomedical waste management among operation room personnel in a tertiary care center. J. Anaesthesiol. Clin. Pharmacol..

[29] Akkajit P., Romin H., Assawadithalerd M. (2020). Assessment of knowledge, attitude, and practice in respect of medical waste management among healthcare workers in clinics. J. Environ. Public Health.

[30] Sachan R., Patel M.L., Nischal A. (2012). Assessment of the knowledge, attitude and practices regarding biomedical waste management amongst the medical and paramedical staff in tertiary health care centre. Int. J. Sci. Res. Public.

[31] Albalushi A.Y., Ullah M., Makhamri A., Alalawi F., Khalid M., Alghafri H. (2018). Knowledge, attitude and practice of biomedical waste management among health care personnel in a secondary care hospital of Al Buraimi Governorate, Sultanate of Oman. Glob. J. Health Sci..

[32] Singh T., Ghimire T.R., Agrawal S.K. (2018). Awareness of biomedical waste management in dental students in different dental colleges in Nepal. Biomed. Res. Int..

[33] Bokhoree C., Beeharry Y., Makoondlall-Chadee T., Doobah T., Soomary N. (2014). Assessment of environmental and health risks associated with the management of medical waste in mauritius. APCBEE Procedia.

[34] Kumar R., Samrongthong R., Shaikh B.T. (2013). Knowledge, attitude and practices of health staff regarding infectious waste handling of tertiary care health facilities at metropolitan city of Pakistan. J. Ayub. Med. Coll. Abbottabad..

[35] Dona D., Giaquinto C., Baraldi E., Biffi A., Gamba P., Saieva A.M., Antoniello L., Costenaro P., Masiero S., Sainati L. (2020). COVID-19 Pandemic: Perspective of an Italian tertiary care pediatric center. Healthcare.

